# Age-specific reproduction in female pied flycatchers: evidence for asynchronous aging

**DOI:** 10.1007/s00442-021-04963-2

**Published:** 2021-06-26

**Authors:** Rémi Fay, Pierre-Alain Ravussin, Daniel Arrigo, Jan A. C. von Rönn, Michael Schaub

**Affiliations:** 1 Swiss Ornithological Institute, Seerose 1, CH–6204 Sempach, Switzerland; 2 Rue du Theu 12, CH-1446 Baulmes, Switzerland; 3 Hofmattenstrasse 12, CH-2560 Nidau, Switzerland

**Keywords:** Age-related variation, Aging pattern, Breeding success, *Ficedula hypoleuca*, Nest success, Senescence, Selective appearance and disappearance

## Abstract

Age-related variation in reproductive performance is central for the understanding of population dynamics and evolutionary processes. Our understanding of age trajectories in vital rates has long been limited by the lack of distinction between patterns occurring within- and among-individuals, and by the lack of comparative studies of age trajectories among traits. Thus, it is poorly understood how sets of demographic traits change within individuals according to their age. Based on 40 years of monitoring, we investigated age-related variation in five reproductive traits in female pied flycatchers (*Ficedula hypoleuca*) including laying date, clutch size, brood size, nest success (probability that a nest produces at least one chick) and egg success of successful nests (proportion of eggs resulting in a chick). We disentangled within- from among-individual processes and assessed the relative contribution of within-individual age-specific changes and selective appearance and disappearance. Finally, we compared the aging pattern among these five reproductive traits. We found strong evidence for age-specific performance including both early-life improvement and late-life decline in all reproductive traits but the egg success. Furthermore, the aging patterns varied substantially among reproductive traits both for the age of peak performance and for the rates of early-life improvement and late-life decline. The results show that age trajectories observed at the population level (cross-sectional analysis) may substantially differ from those occurring at the individual level and illustrate the complexity of variation in aging patterns across traits.

**Supplementary Information:**

The online version contains supplementary material available at 10.1007/s00442-021-04963-2.

## Introduction

The continuous change of reproductive performance with age is a fundamental characteristic of most organisms (Clutton-Brock 1988; Newton 1989). The manner in which individual reproductive performance changes from birth to death is important for the understanding of population dynamics and life-history evolution (Stearns 1992). Reproductive performance typically increases in early life, reaches a plateau at intermediate ages and declines at old ages (Clutton-Brock 1988; Newton 1989), but there are differences among species in this general pattern (Jones et al. 2014). The early-life improvement is primarily due to the progressive relaxation of constraints as a result of growth and acquired experience whereas the late-life decline is due to senescence (Forslund and Pärt 1995).

Senescence is defined as the decline in fitness components with increasing age (Rose 1991) and has been extensively studied. Theoreticians have proposed several explanations for the evolution of senescence (Medawar 1952; Williams 1957; Hamilton 1966; Kirkwood and Rose 1991) which have been subsequently supported by empirical studies (e.g.,Charlesworth and Hughes 1996; Gustafsson and Pärt 1990; Stearns et al. 2000). While current evolutionary theories explain the presence of senescence, they provide little basis for the understanding of the variability in aging patterns across different fitness components. Although it is expected that the strength of natural selection against the late-life decline in a given phenotypic trait depends on how this trait affects fitness (Williams 1957), these theories did not formally predict whether senescence acting on different traits is synchronous, i.e., shows the same age-related pattern (Hayward et al. 2015). Previous studies showed that asynchrony in the age-related decline of several phenotypic traits is widespread (Nussey et al. 2009; Hayward et al. 2015; Zhang et al. 2015; Cooper et al. 2021). For instance, in the common lizard *Zootoca vivipara*, adult female survival declines from age 2 while fecundity increases until the age of 4 years (Massot et al. 2011). In contrast, in the wandering albatross *Diomedea exulens*, survival senescence proceeds reproductive senescence. For this species, the probability to successfully rear a fledgling decreases continuously from age 15 to 20 onwards whereas survival declines only after age 30 (Froy et al. 2013; Pardo et al. 2013). Although studies about aging on several traits have been accumulating over the last decade, more empirical data on a larger range of species are needed to advance our understanding of the variability of aging patterns across fitness component and life-history strategies (Bouwhuis and Vedder 2017).

The study of age-related variation in phenotypic traits has faced analytical challenges for a long time (Forslund and Pärt 1995; Reid et al. 2003; Nussey et al. 2008). Indeed, when following individuals over time, the progressive appearance or disappearance of individuals with contrasting phenotypes may change the composition of a cohort as it ages. For instance, individuals with high reproductive performance could be overrepresented in older age classes because survival and reproductive abilities could be positively correlated (Vedder and Bouwhuis 2018). As a consequence, the average age trajectory observed at the population level (cross-sectional) may differ from the average individual age trajectory (Vaupel and Yashin 1985). Longitudinal datasets are needed to disentangle selective appearance and disappearance from within-individual age trajectories (van de Pol and Verhulst 2006). However, appropriate statistical methods have been popularized relatively recently and many studies have inferred individual age trajectories from average population patterns without controlling for selective processes. Although the inadequacy of this practice is now clearly recognized (Nussey et al. 2008), we still poorly understand how population-level age trajectories reflect the average within-individual age trajectories rather than changes in the phenotypic composition of the population (but see Hayward et al. 2013; Zhang et al.- 2015). As a consequence, it is unclear to what extent age-related patterns published in earlier studies are comparable to more recent studies that accounted for selective processes.

Pied and collared flycatchers (*Ficedula hypoleuca* and *F. albicollis*) are among the most studied bird species in Europe. Age trajectories in reproductive performance have been investigated in several populations, but most previous studies provided only age trajectories at the population level without controlling for selective appearance or disappearance (Sternberg 1989; Gustafsson and Pärt 1990; Lundberg and Alatalo 1992; Sanz and Moreno 2000; Sendecka 2007). Recent studies are more in line with current statistical standards for the investigation of age trajectories (Evans and Sheldon 2013; Potti et al. 2013; Spagopoulou et al. 2020). However, Evans et al. (2011) likely used an inappropriate equation for the within-centering method as Fay et al. 2020a recently pointed out. Potti et al. (2013) estimated age-specific clutch size and the number of fledged young per egg, but considered groups of females according to their ornamentation. Spagopoulou et al (2020) used a single reproductive trait (number of recruits) which integrates reproductive success, post-fledging survival, dispersal and recruitment probability. None of these studies have run a model selection procedure to evaluate the shape of age-related variation in reproductive performance. Thus, despite the numerous studies on pied and collared flycatchers, we have still a poor understanding of the average within-individual age-specific reproductive performance in these species.

In this paper, we investigate age trajectories in female pied flycatchers for five reproductive traits: laying date, clutch size, brood size, nest success and egg success. All these reproductive traits contribute to the overall fecundity and thus are strongly related to individual fitness. They also allow the investigation of both physiological and behavioral aspects of fecundity. While laying date, clutch size and brood size are expected to be related to female condition (Bolton et al. 1993; Descamps et al. 2011), nest and egg success are more likely to be related to the female’s choice of nest location and her feeding effort (Ringsby et al. 2009; Horie and Takagi 2012). Measuring multiple components of fecundity may also reveal contrasting age-specific patterns in reproductive traits that would not be detected otherwise. Here, we describe both population and individual level average age trajectories of these five traits. We control for annual variation in the environment, fixed among-individual variation, and for selective appearance and disappearance. Specific aims of the study are (i) the estimation of average within-individual age trajectories in five reproductive traits including both early-life improvement and late-life decline, (ii) disentangling within- from among-individual effects and the assessment of their relative contribution and finally (iii) the investigation of the variability of aging patterns among the five reproductive traits.

## Material and methods

### Studied species and data collection

Pied flycatchers are small (∼13 g), migratory, cavity-nesting passerines which typically breed in woodlands. This species easily accepts nest boxes and even seems to prefer them over natural cavities (Lundberg and Alatalo 1992). Pied flycatchers are typically single brooded with biparental care. The female incubates alone and broods the chicks until the age of 7 days but both parents usually contribute to feeding the nestlings. Nestlings were ringed with a standard aluminum ring at an age of 12 days. Fledging occurs at an age of 14–17 days. Individuals typically start to reproduce when 1 year old, but some may start their reproductive life at older ages (Lundberg and Alatalo 1992).

The data were collected from two nest box breeding populations in Switzerland in the north of canton of Vaud (46°47′ N/6°32′ E and 46°50′ N/6°42′ E). Since the beginning of their monitoring which started in 1980 and 1989, respectively, both populations have been followed annually until 2019. In total, an annual average of 259 nest boxes were available (range 34–406) supporting the reproduction of 36 pairs (range 13–64). These two populations are about 15 km apart and both are distant from other populations by about 50 km. The isolation results in high site fidelity of individuals (mean adult apparent survival > 0.5, Ravussin et al. 2007) making these two populations highly valuable for the study of age-dependent trajectories. Nest boxes were checked at least once a week and laying date, clutch size, nest success (i.e., the probability that a clutch produces at least one chick reaching the ringing age), brood size of a successful nest (i.e., the number of chicks reaching the ringing age per successful nest) and the egg success of a successful nest (i.e., the probability that an egg produces a chick reaching the ringing age given that the nest was successful) were recorded. Females were mostly caught when incubating and males when feeding the nestlings. As extra-pair paternity as well as polygyny occurs in our population, data on age-specific reproductive performance are less accurate for males than for females. In addition, it is easier to catch incubating females than males, rendering female life-history trajectories more complete. We therefore focused our analyses on females only. The age was known for individuals that were ringed during their year of birth (*n* = 364), and unknown for all birds captured first as an adult (*n* = 295). Because few individuals of known age lived longer than 6 years (*n* = 12), ages higher or equal to 6 were pooled to a common age class. Age-specific data include 604 laying dates from 338 individuals, 624 clutch sizes from 353 individuals, 554 brood sizes from 313 individuals, 654 nest successes/failures from 364 individuals and 3509 egg successes/failures from 306 individuals. Observed ages at first and last reproduction varied between 1 to 5 and 1 to 8, respectively.

### Data analysis

#### Modeling age trajectories at the individual level

We used a linear mixed effect model with a Normal error to analyze laying date, clutch size and brood size of known aged females (van de Pol and Verhulst 2006; Kéry and Schaub 2012). Normal regression models were preferred over Poisson regression models because only the former fitted the observed data well (see Appendix S1). The distribution of brood size was unimodal, because it was conditioned on nest success, and hence similarly well suited for a model with a Normal error distribution as the other reproductive traits. The applied linear model is given by the following equations:

$$\Omega _{{i, t}} \sim N\left( {\omega _{{i,t}} , \sigma ^{2} _{\gamma } } \right)$$

$$\omega _{{i,t}} = \alpha _{{{\text{Age}}_{i,t}}} + \beta _{1} *AFR_{i} + \beta _{2} *ALR_{i} + \gamma _{{{\text{pop}}_{i} }} + \varepsilon _{t}^{{{\text{year}}}} + \varepsilon _{i}^{{{\text{ind}}}}$$

where ${\varepsilon }_{t}^{\text{year}}\sim N(0,{\sigma }_{\text{year}}^{2})$ and ${\varepsilon }_{i}^{\text{ind}}\sim N(0,{\sigma }_{\mathrm{ind}}^{2})$ where ${\Omega }_{i,t}$ is the standardized (z-transformed) reproductive trait of female *i* in year *t* (i.e., laying date, clutch size or brood size). *α* accounts for the age effect (see paragraph Modelling age effects and model selection). ${pop}_{i}$ is a Boolean variable controlling for the population effect and $\gamma$ is the difference in trait $\Omega$ between the two populations. *AFR* and *ALR* are the ages at which female *i* was first and last known to reproduce and ${\beta }_{1}$ and ${\beta }_{2}$ are the respective estimates of selective appearance and disappearance. The individual random effect (${\varepsilon }_{i}^{\text{ind}}$) accounts for the among-individual heterogeneity that is not explained by selective appearance or disappearance and for the non-independence among multiple observations over an individual’s life-history. The random year effect (${\varepsilon }_{t}^{\text{year}}$) accounts for environmental stochasticity. Finally, ${{\sigma }^{2}}_{\gamma }$ is the residual variance.

Data of females of unknown age were jointly analyzed with the data of known aged females to improve the estimates of year random effect. For females of unknown age, we used the following linear model:

$$\Omega _{{i, t}} \sim N\left( {\omega _{{i,t}} , \sigma ^{2} _{{\gamma ^{\prime } }} } \right)$$

$$\omega _{{i,t}} = \eta + \gamma _{{{\text{pop}}_{i} }} + \varepsilon _{t}^{{{\text{year}}}} + \varepsilon _{i}^{{{\text{ind}}}}$$

where ${\varepsilon }_{t}^{\text{year}}\sim N(0,{\sigma }_{\text{year}}^{2})$ and ${\varepsilon }_{i}^{\text{ind}}\sim N(0,{\sigma }_{\text{ind}}^{2}).$ *η* is the mean reproductive trait value of females of unknown age and ${{\sigma }^{2}}_{\gamma ^{\prime}}$ is the residual variance. All the other parameters are the same as those from the equations of known aged females.

We modeled nest success of females of known age with a generalized linear mixed effect model with a Bernoulli error distribution:

$${\mathrm{N}\mathrm{S}}_{i,t}\sim \text{Bern}({\omega }_{i,t})$$

$${\text{logit}}\left( {\omega _{{i,t}} } \right) = \alpha _{{\text{Age}_{i,t}}} + \beta _{1} {\text{*AFR}}_{i} + \beta _{2} {\text{*ALR}}_{i} + \gamma _{{{\text{pop}}_{i} }} + \varepsilon _{t}^{{{\text{year}}}}$$

where ${\varepsilon }_{t}^{\text{year}}\sim N(0,{\sigma }_{year}^{2})$. NS_i,t_ indicates whether the brood of female *i* in year *t* was successful (${\mathrm{N}\mathrm{S}}_{i,t}=1$) or failed (${\mathrm{N}\mathrm{S}}_{i,t}=0$). All parameters and explanatory variables have the same definitions as in the previous model. Note that we did not add an individual random effect because this parameter failed to be estimated correctly. Individual random effects were bimodally distributed with one mode corresponding to individuals that had failed at least once and another for those that never failed. Keeping this overfitted random effect would strongly bias the estimates of other parameters. As for the previous reproductive traits, the females of unknown age were jointly analyzed to improve the estimates of random effects.

We analyzed the egg success of known age females with a generalized linear mixed model with a binomial error distribution:

$$\text{ES}_{{i,t}} \sim \text{Bin}(n_{{i,t}} , p_{{i,t}} )$$

$${\text{logit}}\left( {p_{{i,t}} } \right) = \alpha _{\text{Age}_{i,t}} + \beta _{{{\text{AFR}}}} {\text{*AFR}}_{i} + \beta _{{{\text{ALR}}}} {\text{*ALR}}_{i} + \gamma _{{{\text{pop}}_{i} }} + \varepsilon _{i}^{{{\text{ind}}}}$$

where ${\varepsilon }_{t}^{year}\sim N(0,{\sigma }_{\text{year}}^{2})$ and ${\varepsilon }_{i}^{\text{ind}}\sim N(0,{\sigma }_{\text{ind}}^{2})$. ES_i,t_ indicates the number of chicks reaching the ringing age for brood of female *i* in year *t*. ${n}_{i,t}$ is the number of eggs in the reproductive attempt of female *i* in year *t* and ${p}_{i,t}$ is the probability that an egg produces a chick reaching the ringing age. All other parameters and explanatory variables have the same definition as in the previous models. Females of unknown age were jointly analyzed to improve the estimates of random effects.

#### Modeling age trajectories at the population level

To investigate age-related variation in reproductive traits at the population level, we modified the generalized mixed models described for the individual level by removing parameters that account for selective appearance and disappearance and the individual random effect. Thus, to model laying date, clutch size and brood size of the known aged females we used the following regression model:

$$\Omega _{{i,t}} \sim N\left( {\omega _{{i,t}} , \sigma ^{2} _{\gamma } } \right)$$

$$\omega _{{i,t}} = \alpha _{\text{Age}_{i,t}} + \gamma _{{{\text{pop}}_{i} }} + \varepsilon _{t}^{{{\text{year}}}}$$

where ${\varepsilon }_{t}^{\text{year}}\sim N(0,{\sigma }_{\text{year}}^{2})$. The adults of unknown age were jointly analyzed to improve the estimates of random effects similarly to what had been presented for the individual level. Now the equations are:

$$\Omega _{{i,t}} \sim N\left( {\omega _{{i,t}} , \sigma ^{2} _{{\gamma ^{\prime } }} } \right)$$

$$\omega _{{i,t}} = \eta + \gamma _{{{\text{pop}}_{i} }} + \varepsilon _{t}^{{{\text{year}}}}$$

where ${\varepsilon }_{t}^{\text{year}}\sim N(0,{\sigma }_{\text{year}}^{2})$. For the remaining reproductive traits, we preceded in the same way by removing effects of selective appearance and disappearance and of the individual random effect from the generalized mixed model described for the individual level.

#### Modelling age effects and model selection

We investigated age effects (*α*) by comparing a variety of different models. The set of these candidate model included a model with age fitted as a categorical variable (${{\alpha }_{\text{Age}}}_{i,t}$) which correspond to the general model presented above, a model without any age effect (${\alpha }_{cst}$) and models with different continuous functions of age. These included linear (${\alpha }_{lin}$) and quadratic (${\alpha }_{qua}$) age effects as well as single (${\alpha }_{thrX}$) and double thresholds (${\alpha }_{thrX-X}$) (age *X* varying between 2–5). Thus, the set of candidate models comprised 14 models in total. Models were compared by the widely applicable information criterion (WAIC; Watanabe 2010), a fully Bayesian information criterion used to measure the predictive accuracy of a model. Model averaging was performed across models that had a ΔWAIC < 2.

#### Estimation of the rates of early-life improvement and late-life decline

Based on the results of the model selection, we identified for each reproductive trait the individual and the population level age of peak performance, the value at peak performance, and the rates of improvement (i.e., from age 1 to peak age), and of decline (i.e., from peak age to age 6 +). To compare the average within-individual rates of improvement and decline among traits, we standardized (z-transformation) age-specific estimates from models with age fitted as a categorical variable (categories being age 1, 2, 3, 4, 5 and 6 +) and estimated the slopes before and after the identified age of peak performance with a linear regression model.

#### Estimation of the relative contribution of within-individual age effects, selective appearance and disappearance to individual age trajectories

We calculated the relative proportion of the variance in the observed data that is explained by within-individual age effects, selective appearance and disappearance effects, respectively (R^2^). First, we computed R^2^ by adapting the conventional formula to our response variable based on the estimates from the best supported model:

$$R^{2} = \frac{{{\text{SSR}}}}{{{\text{SSTO}}}} = \frac{{\sum\limits_{{i = 1}}^{n} {(\hat{X}_{{i,t,j}} - \bar{X}_{{i,t}} )^{2} } }}{{\sum\limits_{{i = 1}}^{n} {(X_{{i,t}} - \bar{X}_{{i,t}} )^{2} } }}$$

Here, ${X}_{i,t}$ is the observed performance of individual *i* in year *t* in trait *X*. ${\widehat{X}}_{i,t,j}$ is the predicted performance of individual *i* in year *t* for trait *X* when including only the effect of component *j* (i.e., within-individual age effects, selective appearance effect, or the selective disappearance effect). We removed the effect of the other components from the model using their average values. For instance, to compute R^2^ for the within-individual age effect, we used the predicted performances including only the effect of age by applying the following equation:

$$\hat{X}_{{i,t,{\text{Age}}}} = \alpha _{{i,t}} + \beta _{1} {\text{*}}\overline{{{\text{AFR}}}} + \beta _{2} {\text{*}}\overline{{{\text{ALR}}}} + \gamma _{{{\text{pop}}_{i} }} + \varepsilon _{t}^{{{\text{year}}}} + \varepsilon _{i}^{{{\text{ind}}}}$$

where $\stackrel{-}{AFR}$ and $\stackrel{-}{ALR}$ are the average ages at which the individual was first and last known to reproduce. $\bar{X} _{i,t}$ is the predicted performance after removing all the effects of age and selective processes using their average values which corresponds to the following equation:

$$\bar{X}_{{i,t}} = \alpha _{{\overline{\text{Age}} }} + \beta _{1} *\overline{\text{AFR}} + \beta _{2} *\overline{\text{ALR}} + \gamma _{{\text{pop}}_{i}} + \varepsilon _{t}^{\text{year}} + \varepsilon _{i}^{\text{ind}}$$

Once R^2^ has been computed for each component *j*, the relative contributions were derived by dividing each estimated ${R}_{j}^{2}$ by the sum of all estimates, i.e., $\sum _{j=1}^{3}{R}_{j}^{2}$.

### Parameter estimation

We used the Bayesian approach and Markov chain Monte Carlo (MCMC) simulation for parameter estimation. We specified weakly informative priors for all estimated parameters. We used the uniform distribution on the interval [− 5, 5] as priors for the regression parameters of standardized traits (laying date, clutch size and brood size) and the uniform distribution on the interval [− 20, 20] for the regression parameters of the other traits (egg success and nest success). We used the uniform distribution on the interval [0, 5] for the standard deviations of the random effects. The analysis was conducted in JAGS (Plummer 2003) via the R package jagsUI (Kellner 2016). See appendix S2 & S3 for examples of JAGS code. Posterior summaries from three MCMC chains were based on 50,000 iterations after a burn-in of 10,000 and a thinning interval of 10. We confirmed parameter convergence by visual inspection of the trace plots and using the Gelman–Rubin statistic (Brooks and Gelman 1998). All the R-hat values were below 1.1 supporting convergence.

## Results

We first present the age trajectories of average within-individual variation in the five reproductive traits and secondly present the relative importance of selective appearance and disappearance on these patterns. Finally, we compare the aging patterns of the five reproductive traits.

### Average within-individual age trajectories in reproductive performance

**Fig. 1:**
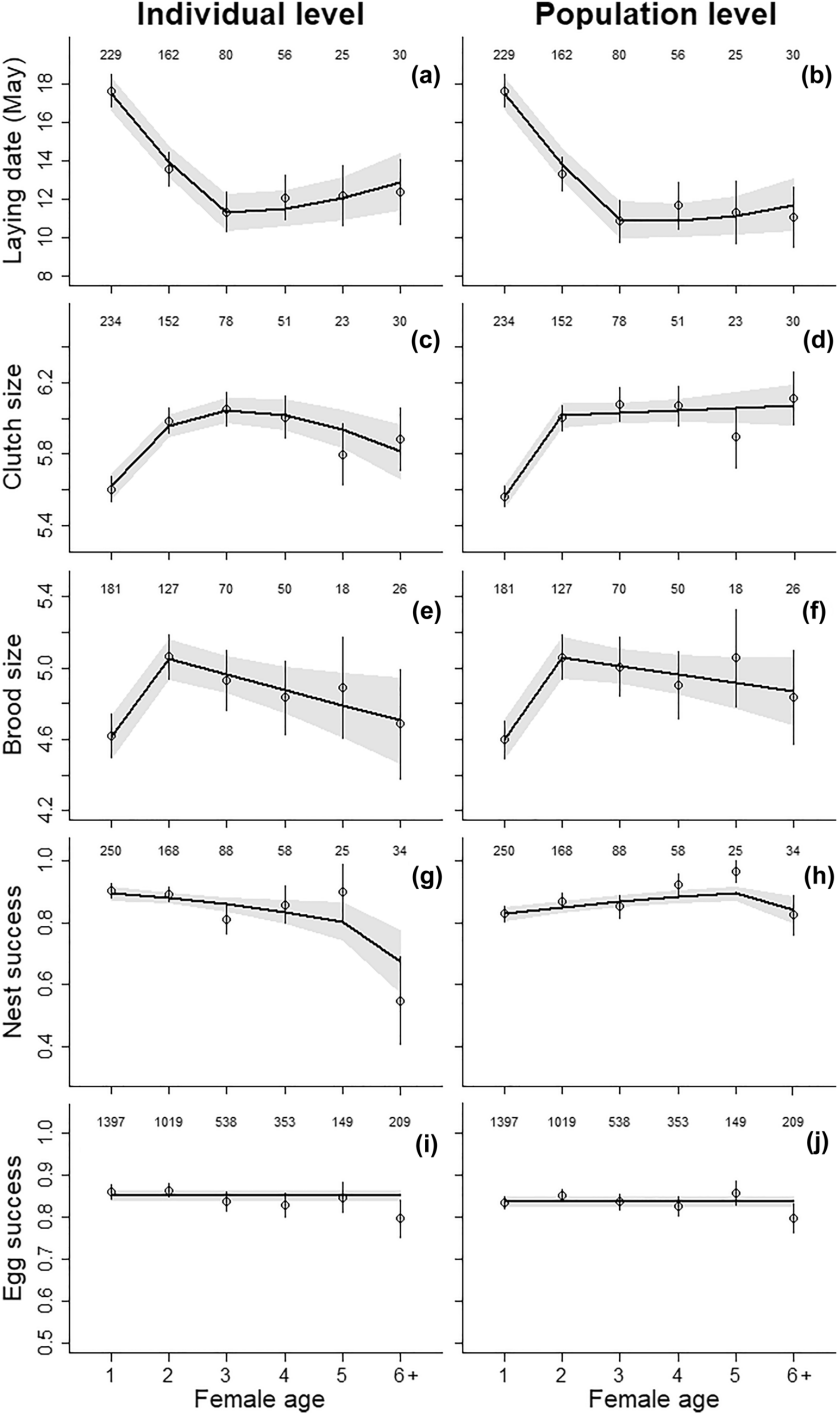
Estimated average age trajectories in laying date (**a**, **b**), clutch size (**c**, **d**), brood size (**e**, **f**), nest success (**g**, **h**) and egg success (**i**, **j**) in females pied flycatchers at the individual and the population level. Points and vertical bars show estimates from a model with age fitted as a categorical variable, ± standard error. The solid black lines show the aging patterns predicted by model averaging over the best models (ΔWAIC < 2), with the grey-shaded areas showing model averaged standard errors around the average predictions. Numbers on the top refer to sample sizes (number of individuals of a given age)

Model selection supported within-individual age effects in all traits with the only exception of the egg success (Table 1). Laying date decreased by 6 days from age 1 to 3 and then continuously increased until age 6 + by a total of 1.5 days (Fig. 1, Table 2). Clutch size showed the same general pattern with a peak of performance of six eggs at age 3. One-year-old and 6 +-year-old females laid clutches that were respectively 0.4 and 0.2 eggs smaller than those of 3-year-old females. Model selection for clutch size provided support for five models with different age functions (ΔWAIC < 2, Table 1). Brood size was best described by a single threshold model with a break point at age 2. Early-life improvement and late-life decline were roughly similar to those of clutch size. The best model for nest success suggested a slight continuous decrease until age 5 and then a strong decrease. Although the null model was within ΔWAIC = 1.1 from the best model, the relative weight of the top supported models (i.e., cst, lin, Thr3) showed that the decrease with age was three times more likely than that there was no age variation (0.14 vs 0.41, Table 1). However, this result was strongly affected by the drop of performance after age 5. Finally, model selection suggested that egg success showed no relationship with age. The probability that an egg produced a chick reaching the ringing age in a successful nest was 0.85 (Fig. 1).

**Table 1:** Model selection for average within individual age trajectories of five reproductive traits in female pied flycatchers

Age (α)	Laying date		Clutch size		Brood size		Nest success		Egg success	
	ΔWAIC	w_i_	ΔWAIC	w_i_	ΔWAIC	w_i_	ΔWAIC	w_i_	ΔWAIC	w_i_
Cst	62.8	0.00	23.6	0.00	6.9	0.02	1.1	0.14	**0.0**	**0.53**
Age	8.2	0.01	7.0	0.01	9.3	0.01	8.9	0.00	13.3	0.00
Lin	34.1	0.00	22.4	0.00	10.3	0.00	0.7	0.17	2.4	0.16
Qua	1.2	0.20	**0.0**	**0.29**	3.8	0.09	2.4	0.07	4.5	0.06
Thr2	5.7	0.02	1.1	0.17	**0.0**	**0.57**	2.9	0.06	4.1	0.07
Thr3	**0.0**	**0.37**	1.7	0.13	6.1	0.03	3.9	0.04	5.3	0.04
Thr4	16.0	0.00	8.2	0.00	11.4	0.00	2.6	0.06	5.6	0.03
Thr5	23.9	0.00	17.2	0.00	9.1	0.01	**0.0**	**0.24**	4.5	0.06
Thr2-3	1.1	0.22	1.2	0.16	4.7	0.05	6.4	0.01	8.3	0.01
Thr2-4	5.4	0.03	1.5	0.14	3.2	0.11	5.1	0.02	8.5	0.01
Thr2-5	4.4	0.04	4.0	0.04	3.7	0.09	3.1	0.05	8.9	0.01
Thr3-4	4.0	0.05	4.2	0.04	8.5	0.01	4.5	0.03	8.6	0.01
Thr3-5	3.6	0.06	5.5	0.02	8.7	0.01	2.4	0.07	6.4	0.02
Thr4-5	18.8	0.00	12.5	0.00	13.8	0.00	3.9	0.03	9.1	0.01

ΔWAIC is the difference in WAIC (widely applicable information criterion) between a given model and the model with the lowest WAIC (in bold). w_i_ is the weight of evidence in favor of model i given the set of candidate models. Model notation: Cst = no age effect, Age = age fitted as a categorical variable, Lin = linear effect of age, Qua = quadratic effect of age, ThrX = single threshold model with a breaking point at age X, ThrX-X′ = double threshold model with breaking points at age X and X’

**Table 2:** Comparison of age-related change in reproductive traits at the population (pop) and individual (ind) level in female pied flycatchers

Trait (unit)	Observation level	Peak age	Peak performance	Improvement effect size	Senescence effect size
Laying date (days May)	pop	4	10.90	− 6.59	0.81
	ind	3	11.35	− 6.11	1.53
Clutch size (eggs)	pop	6+	6.07	0.51 (9%)	0
	ind	3	6.04	0.43 (8%)	− 0.23 (4%)
Brood size (chicks)	pop	2	5.06	0.46 (10%)	− 0.19 (4%)
	ind	2	5.05	0.44 (10%)	− 0.34 (7%)
Nest success (probability)	pop	5	0.90	0.07 (8%)	− 0.05 (6%)
	ind	1	0.90	0	− 0.22 (24%)
Egg success (probability)	pop	–	0.84	0	0
	ind	-–	0.85	0	0

Shown are the ages of peak performance, model averaged estimates of peak performance, and effect sizes of improvement and decline. Effect sizes are expressed as changes in performance between age 1 and the peak age and between peak age and age 6+ , respectively

### Disentangling within- and among-individual effects

**Fig. 2:**
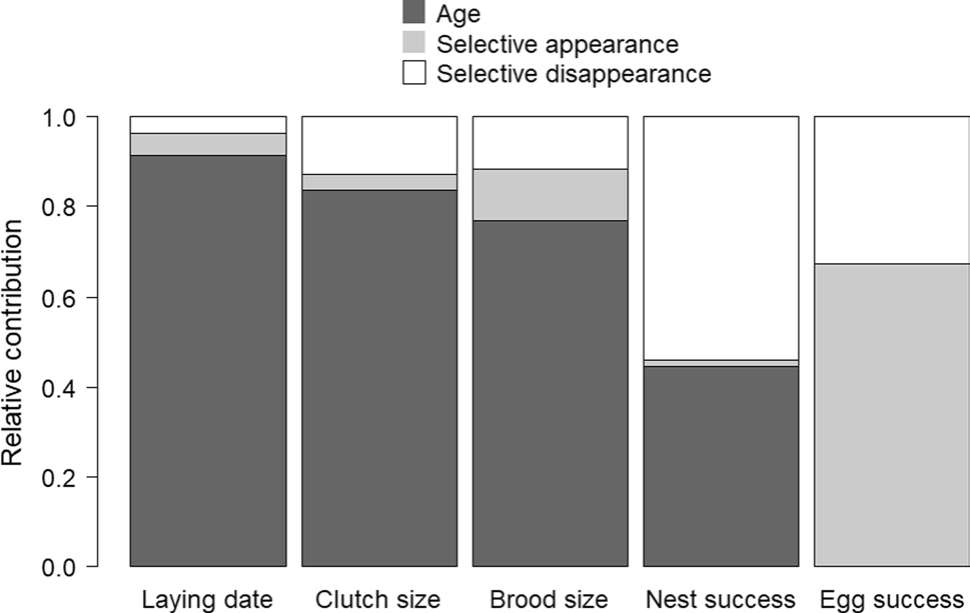
Relative contribution of the within-individual age effect, selective appearance and selective disappearance to the variation of five reproductive traits in female pied flycatchers

Although the posterior distributions for selective appearance and disappearance overlapped zero in most cases, estimates for AFR were consistently negative, and estimates for ALR were consistently positive (Table 3)*.* We noted that evidence for selective disappearance for nest success was strong with the 95% credible interval not overlapping zero. These results suggest that individual performances were negatively correlated with recruitment age and positively correlated with longevity. The relative importance of the selective processes relative to the effect of age varied among traits. For laying date, clutch size and brood size, most of the variability in the observed data was explained by within-individual age-related variation (relative contribution varying between 77–91%, Fig. 2). In contrast, for nest success, selective disappearance explained about the same amount of variability as the within-individual age effect.

**Table 3:** Estimated selective appearance and disappearance effects on reproductive traits in pied flycatcher females

Trait	Slope	95% CRI	*f*	Effect size
Selective appearance				
Laying date	0.062	[− 0.019,0.143]	0.936	0.579 (days)
Clutch size	− 0.033	[− 0.123,0.056]	0.770	− 0.028 (egg)
Brood size	− 0.072	[− 0.166,0.022]	0.934	− 0.098 (chick)
Nest success	− 0.035	[− 0.290,0.227]	0.609	− 0.004 (prob)
Egg success	0.132	[− 0.035,0.303]	0.938	0.016 (prob)
Selective disappearance				
Laying date	− 0.023	[− 0.082,0.036]	0.782	− 0.207 (days)
Clutch size	0.050	[− 0.017,0.117]	0.909	0.045 (egg)
Brood size	0.037	[− 0.035,0.109]	0.847	0.051 (chick)
Nest success	0.503	[0.257,0.779]	1	0.050 (prob)
Egg success	− 0.028	[− 0.109,0.049]	0.758	0.003 (prob)

Given are the mean, the 95% CRI and the proportion of the posterior with the same sign as the mean (*f*). Effect sizes express the change in average performance for each additional year by which recruitment is postponed (i.e., selective appearance) or longevity increased (i.e., selective disappearance). Estimates are from the best supported model (Table 1)

The age-related variation in reproductive performance observed at the population level tended to differ from those occurring at the individual level (Fig. 1, Table S1). While the shapes of the general patterns were similar for some traits (laying date, brood size, egg success), others showed substantial differences (clutch size, nest success). For the latter, age of peak performance differed with mismatches from 1 to 4 years depending on the reproductive trait. Furthermore, the early-life improvements observed at population level were slightly overestimated (by 0–19% for laying date, brood size and clutch size) and the late-life declines clearly underestimated (by 44–100% excluding egg success) (Table 2).

### Synchrony of average within-individual age trajectories among reproductive traits

**Fig. 3:**
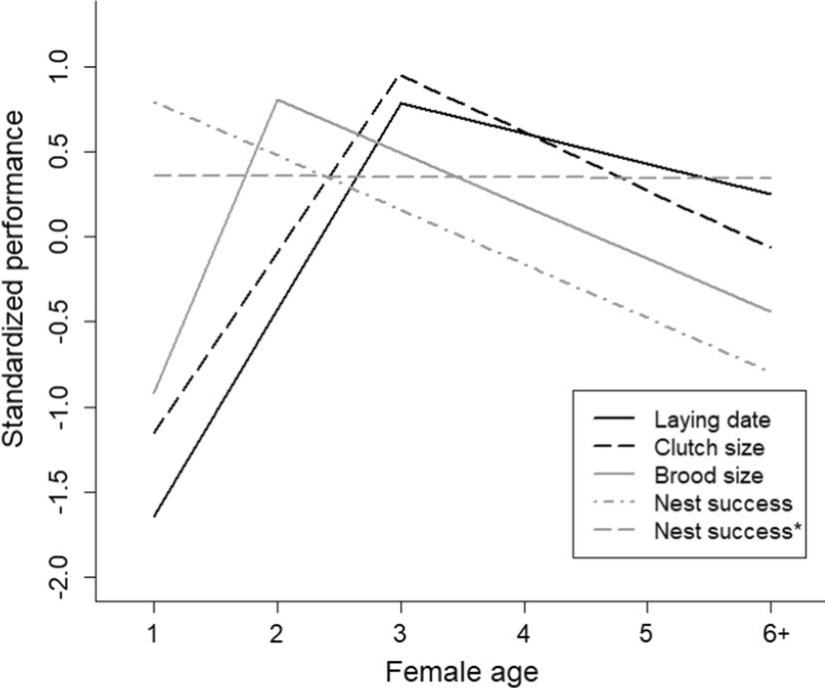
Standardized estimates of improvement and senescence rates in four reproductive traits in female pied flycatchers. For nest success, senescence was re-estimated ignoring the last age class due to the strong change occurring at this age (Nest success*)

Among the common set of investigated models, none was selected as the best model twice suggesting that aging in reproductive traits were largely asynchronous (Figs. 1 and 3, Table 1). Early-life improvement was clearly supported for laying date, clutch size and brood size but was absent for nest success and egg success. The improvement rate was stronger in brood size than in laying date and clutch size (Fig. 3, Table 4). In all traits for which there was evidence of a late-life decline, the onset of the decline varied from age 1 to 3 (Table 2). The rate of decline was 1.8 time less important in the laying date than in the other reproductive traits. The average decline rate was similar for clutch size, brood size and nest success (Fig. 3, Table 4). However, the decline rate estimated for nest success must be interpreted with care as it was highly affected by the strong decrease from age 5 to 6 + (Table 1). The decline in nest success would be negligible (− 0.005) if it is estimated from age 1 to 5 only.

**Table 4:** Standardized estimates of improvement and decline rates

Trait	Improvement slope	95% CRI	Decline slope	95% CRI
Laying date	1.22	[1.21,1.22]	− 0.18	[− 0.17,− 0.19]
Clutch size	1.06	[1.05,1.08]	− 0.34	[− 0.33,− 0.35]
Brood size	1.72	[1.69,1.76]	− 0.32	[− 0.31,− 0.33]
Nest success	–	–	− 0.31	[− 0.30,− 0.32]
Nest success*	–	–	− 0.01	[− 0.01,− 0.02]

The estimated slopes are the rate of increase and decrease before and after the age of peak performance (Table 1). Slopes are estimated by fitting threshold models to standardized age-specific means of all traits. For nest success, senescence was re-estimated ignoring the last age class due to the strong change in performance occurring at this age (Nest success*)

## Discussion

Reproductive performance varied within individuals in relation to their age in four out of five investigated traits. The aging patterns varied substantially among reproductive traits both for the age of peak performance and for the rate of early-life improvement and late-life decline. Furthermore, individuals that started reproducing at an early age and those with higher longevity generally performed better than the average, regardless of their age. Because selective processes were acting, age trajectories observed at the population level (cross-sectional) generally differed from the average within-individual age trajectories.

### Reproductive aging and synchrony

At the individual level, laying date, clutch size and brood size peaked when females were 2–3 years old, which corresponds to results of previous studies in female flycatchers (Gustafsson and Pärt 1990; Potti et al. 2013). Compared to the maximum longevity of pied flycatchers (around 10 years, Fransson et al. 2017), the ages of peak performances were early in life. The improvement of clutch size with age was consistent with previous estimates in pied and collared flycatchers with a difference of 0.5 eggs between first time and intermediate age breeders, but the advancement of the laying date appeared to be stronger in our population with 6 days against 2–4 days in previous studies (population level estimates, Gustafsson and Pärt 1990; Jarvinen 1991). The age of peak performance of reproductive traits at 2–3 years seems to be a common pattern in females of short lived passerines (Eurasian blue tit *Cyanistes caeruleus* Amininasab et al. 2017; Barn swallow *Hirundo rustica* Balbontin et al. 2007; Great tit *Parus major* Bouwhuis et al. 2009; Song sparrow *Melospiza melodia* Keller et al. 2008; House sparrow *Passer domesticus* Schroeder et al. 2012).

Early-life improvement of reproductive competence may partly be due to breeding experience as shown experimentally in pied flycatchers (Cichoń 2003). The early-life improvement in clutch and brood sizes could also be partly a byproduct of the increasing ability of individuals to initiate reproduction earlier. Due to the progressive seasonal degradation of breeding conditions, the advancement of laying date increases breeding success in a large number of birds species (Verhulst and Nilsson 2008) including pied flycatchers (Siikamäki 1998), independently of parental experience. Similarly, the decrease in clutch and brood sizes from age 3 onwards may also partially be explained by progressively later laying dates. However, in contrast to the improvement rate in the early life, the decline rate in laying date was lower than for clutch and brood size. This suggests that the lower ability of older females to initiate a clutch early in the breeding season was not enough to explain the decrease in other reproductive traits, and thus, that an intrinsic decrease in reproductive ability was also occurring. This was confirmed when estimating age-related variation in clutch and brood sizes after controlling for laying date (Fig S1 & S2) and is consistent with the view that reproductive senescence affects all aspects of the reproductive cycle (Lemaître and Gaillard 2017). Nevertheless, our study suggests that not all reproductive components contribute equally to the decrease in fecundity. Although not fully synchronized, decline rates of clutch and brood sizes were similar suggesting that the age-dependent decline in the number of chicks was mainly caused by age-related variation in clutch size. Consistently, once clutch size was controlled for, we found no effect of female age on the egg success, indicating that the ability of a female to incubate eggs and raise chicks remains constant over her entire life. Clutch size variation seems also to explain a substantial part of the age-related variation of brood size in blue and great tits (Dhondt 1989; Auld and Charmantier 2011 but see Bouwhuis et al. 2009).

In contrast to other reproductive traits, nest success was weakly affected by age before a female was 5 years old, but then decreased steeply. Although not unprecedented (e.g., body mass in female Soay sheep (Hayward et al. 2015)), we cannot totally exclude that the abrupt decline in the last age class is be due to sampling variance and thus should be interpreted with caution. It is doubtful that nest success decreases from age 1 to age 2–3 since other breeding parameters strongly improve during this period, suggesting that the reproductive ability of female strongly increases. The weak relationship with age during most of the life-time questions the ability of birds to control nest success, especially under circumstances of artificial nesting sites, such as nest boxes. Accordingly, in our flycatcher population, nest success was strongly affected by external factors such as predation or human disturbance. Owing to the natural stochasticity of these factors and potentially the lack of alternative nesting sites, it might be difficult for pied flycatchers to find nesting sites in the first place and assess nesting site safety at settlement and thus to control nest success. In small passerines, the stochasticity in nest success can be an important process to mask potential age effects in fecundity (Fay et al. 2020b; Mitrus 2004 but see Horie and Takagi 2012; Robertson and Rendell 2001; Pärt 2001). This contrasts with long-lived species for which nest success is more frequently associated with age (Berman et al. 2009; Froy et al. 2017; Murgatroyd et al. 2018; Newton et al. 1981). Because breeding failure is a key component of breeding performance, nest success may affect the overall relationship between age and fecundity (Fay et al. 2020b). Thus, more studies are needed to seek for generality in the critical relationship between nest success and age, especially in short-lived species.

### Selective appearance and disappearance

Individuals that delay recruitment tend to lay clutches later, to have smaller clutches and to raise fewer offspring compared to females of the same age that are recruited earlier. These results suggest that recruitment age is affected by individual quality as found in a previous study in collared flycatchers (Pärt 1995) and in numerous bird and mammal species (Arcese 1989; Lee et al. 2013; Zhang et al. 2015; Fay et al. 2016). We found that for each additional year by which recruitment was postponed, the average annual reproductive performance decreased by 1–5% depending on the trait. However, because recruitment in pied flycatchers is mostly restricted to the first 2 years of life, the effect of selective appearance on the overall age pattern was modest. We also found that all reproductive traits, with the exception of egg success, tended to be positively correlated with the life span supporting the presence of selective disappearance. Thus, the late-life decline was masked at the population level by the progressive disappearance of individuals with lower performance leading to substantial differences with decline observed at the individual level, the latter being the correct measure of aging from an evolutionary perspective (McCleery et al. 2008; Nussey et al. 2008). Selective disappearance was the strongest for nest success, but could be inflated by the potentially higher dispersal of failed breeders (Hoover, 2003, Schaub and van Hirschheydt 2009). Although our population is strongly isolated, permanent emigration may still occasionally occur and breeding failure and disappearance from the population may by partly confounded. The presence of selective effects has been reported in many natural populations suggesting that they are a widespread phenomenon (Reid et al. 2003; van de Pol and Verhulst 2006; McCleery et al. 2008; Zhang et al. 2015; Vedder and Bouwhuis 2018). Our study confirms that failing to account for individual heterogeneity may lead to wrong conclusions about individual age trajectories (Hayward et al. 2013).

### Describing age trajectories and associated difficulties

The inclusion of threshold models in the set of candidate models has strongly improved our ability to describe age-related patterns. If we had not included threshold models, quadratic age effect models would have been preferred for clutch size resulting in an overestimation of the age of the onset of the decline and of the rate of the decline. With one exception, age related variability was better described by threshold than by linear or quadratic models. This result agrees with the few previous studies that used this type of model, showing their usefulness (Berman et al. 2009; Froy et al. 2017; Murgatroyd et al. 2018; Rodríguez-Muñoz et al. 2019). A further advantage of threshold models over polynomial models is their lower sensitivity to the imbalance of sample size according to age. In longitudinal datasets, all sampled individuals contribute to early age estimates but, because of the mortality, only few individuals contribute to late-life estimates. In these cases, estimates of polynomial models are impacted strongly by early-life increase but less by the decline at older ages (McCleery et al. 2008). Thus, age-specific estimates for older age classes could be strongly biased in polynomial models and in particular late-life declines could be overestimated when using quadratic models. Unfortunately, many studies investigating age-patterns considered only polynomial models and did not provide age-specific estimates for a graphical assessment of model fit. More generally, given that there is no biological reason to favor quadratic over threshold models, we strongly encourage that both types of models are used. We also encourage the systematic estimation and presentation of age-specific traits, i.e., age fitted as a categorical variable, and their associated standard errors to compare predictions with more raw data.

## Supplementary Information

Below is the link to the electronic supplementary material.

Supplementary file1 (DOCX 142 kb)

## Data Availability

Should the manuscript be accepted, the data supporting the results will be archived in an appropriate repository.
