# Supplementary material for: Age-specific reproduction in female pied flycatchers: evidence for asynchronous aging
Source: Oecologia. 2021 Jun 26;196(3):723–34. doi: 10.1007/s00442-021-04963-2 (PMC8292251; doi:10.1007/s00442-021-04963-2)

Supporting Information

for

Age-specific reproduction in female pied flycatchers: evidence for asynchronous aging

Rémi Fay*^1^, Pierre-Alain Ravussin^2^, Daniel Arrigo^3^, Jan A. C. von Rönn^1^ and Michael Schaub^1^

^1^ Swiss Ornithological Institute, Seerose 1, CH–6204 Sempach

^2^ Rue du Theu 12, CH-1446 Baulmes, Switzerland

^3^ Hofmattenstrasse 12, CH-2560 Nidau, Switzerland

*corresponding author: [fay.remi@gmail.com](mailto:fay.remi@gmail.com)

Appendix S1: Posterior predictive checks of Normal and Poisson regression models

To assess the goodness-of-fit of Normal and Poisson regression models for clutch size and brood size data, we conducted posterior predictive checks using the Freeman-Turkey statistic as discrepancy measures. We also performed a graphical assessment and computed Bayesian p-values for the discrepancy measures (Gelman et al. 2004).

The next figure shows the posterior predictive checks of a Normal and Poisson regression models for clutch size. The solid line represents the 1:1 line. The test indicates no lack of fit for the Normal regression model (Bayesian *P*-value = 0.52). However, there was a strong discrepancy between simulated and observed data when the Poisson regression model was used (Bayesian *P*-value = 1). Observed data were strongly under-dispersed compared to the expectation of a Poisson distribution.


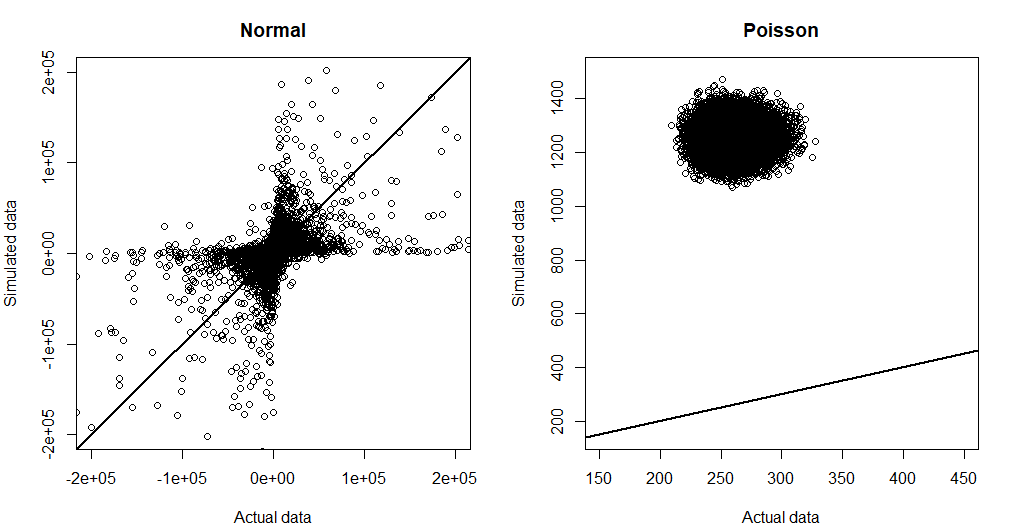


The next figure shows the posterior predictive checks of a Normal and Poisson regression model for brood size. The tests suggest no lack of fit for the Normal regression model (Bayesian P-value = 0.49) and strong lack for the Poisson regression model (Bayesian P-value = 1).


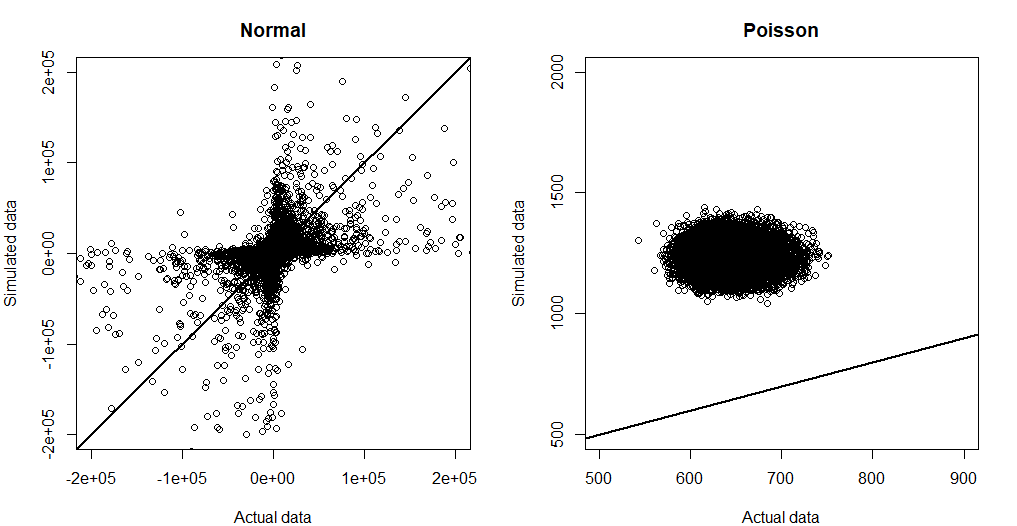


Appendix S2: JAGS code to fit the linear mixed effect model to analyse laying date, clutch size and brood size at the individual and population levels. In this example, models age was fitted as a categorical variable.

#For the individual level

model {

# Priors and constraints

alpha0 ~ dunif(-5,5)

beta2 ~ dunif(-5,5)

beta3 ~ dunif(-5,5)

for (i in 1:age.class){

beta0[i] ~ dunif(-5,5)

}

for (t in 1:n.year){

beta1[t] ~ dnorm(0, tau.year)

}

for (i in 1:n.ID){

eps[i] ~ dnorm(0, tau.ID)

}

gamma ~ dunif(-5,5)

tau.year <- pow(sigma.year, -2) ; sigma.year ~ dunif(0, 5)

tau.ID <- pow(sigma.ID, -2) ; sigma.ID ~ dunif(0, 5)

tau.reg1 <- pow(sigma.reg1, -2) ; sigma.reg1 ~ dunif(0, 5)

tau.reg2 <- pow(sigma.reg2, -2) ; sigma.reg2 ~ dunif(0, 5)

# Regression model

for (i in age.unknown){

Trait[i] ~ dnorm(alpha0 + beta1[year[i]] + eps[ID[i]] + gamma

* pop.ID[i], tau.reg1)

}

for (i in age.known){

Trait[i] ~ dnorm(beta0[AGE[i]] + beta1[year[i]] + eps[ID[i]] +

beta2 * AgeLO[i] + beta3 * AgeFO[i] + gamma * pop.ID[i], tau.reg2)

}

}

#For the population level

model {

# Priors and constraints

alpha0 ~ dunif(-5,5)

for (i in 1:age.class){

beta0[i] ~ dunif(-5,5)

}

for (t in 1:n.year){

beta1[t] ~ dnorm(0, tau.year)

}

gamma ~ dunif(-5,5)

tau.year <- pow(sigma.year, -2) ; sigma.year ~ dunif(0, 5)

tau.reg1 <- pow(sigma.reg1, -2) ; sigma.reg1 ~ dunif(0, 5)

tau.reg2 <- pow(sigma.reg2, -2) ; sigma.reg2 ~ dunif(0, 5)

# Regression model

for (i in age.unknown){

Trait[i] ~ dnorm(alpha0 + beta1[year[i]] + gamma * pop.ID[i],

tau.reg1)

}

for (i in age.known){

Trait[i] ~ dnorm(beta0[AGE[i]] + beta1[year[i]] + gamma *

pop.ID[i], tau.reg2)

}

}

Appendix S3: JAGS code to fit single and double threshold models to analyse laying date, clutch size and brood size at the individual levels.

#Single threshold at age 3

model {

# Priors and constraints

alpha0 ~ dunif(-5,5)

beta0 ~ dunif(-5,5)

beta1 ~ dunif(-5,5)

beta2 ~ dunif(-5,5)

beta5 ~ dunif(-5,5)

beta6 ~ dunif(-5,5)

for (t in 1:n.year){

beta4[t] ~ dnorm(0, tau.year)

}

for (i in 1:n.ID){

eps[i] ~ dnorm(0, tau.ID)

}

gamma ~ dunif(-5,5)

tau.year <- pow(sigma.year, -2) ; sigma.year ~ dunif(0, 5)

tau.ID <- pow(sigma.ID, -2) ; sigma.ID ~ dunif(0, 5)

tau.reg1 <- pow(sigma.reg1, -2) ; sigma.reg1 ~ dunif(0, 5)

tau.reg2 <- pow(sigma.reg2, -2) ; sigma.reg2 ~ dunif(0, 5)

# Covariate

cov1 <- c(1,2,3,4,5,6)

cov2 <- c(0,0,0,1,2,3)

# Regression model

for (i in age.unknown){

Trait[i] ~ dnorm(alpha0 + beta4[year[i]] + eps[ID[i]] + gamma

* pop.ID[i], tau.reg1)

}

for (i in age.known){

Trait[i] ~ dnorm(beta0 + beta1 * cov1[AGE[i]]

+ beta2 * cov2[AGE[i]] + beta4[year[i]] + eps[ID[i]]

+ beta5 * AgeLO[i] + beta6 * AgeFO[i] + gamma * pop.ID[i],

tau.reg2)

}

# Derived quantities

slope1 <- beta1

slope2 <- beta1 + beta2

}

# Double threshold at age 3 and 5

model {

# Priors and constraints

alpha0 ~ dunif(-5,5)

beta0 ~ dunif(-5,5)

beta1 ~ dunif(-5,5)

beta2 ~ dunif(-5,5)

beta3 ~ dunif(-5,5)

beta5 ~ dunif(-5,5)

beta6 ~ dunif(-5,5)

for (t in 1:n.year){

beta4[t] ~ dnorm(0, tau.year)

}

for (i in 1:n.ID){

eps[i] ~ dnorm(0, tau.ID)

}

gamma ~ dunif(-5,5)

tau.year <- pow(sigma.year, -2) ; sigma.year ~ dunif(0, 5)

tau.ID <- pow(sigma.ID, -2) ; sigma.ID ~ dunif(0, 5)

tau.reg1 <- pow(sigma.reg1, -2) ; sigma.reg1 ~ dunif(0, 5)

tau.reg2 <- pow(sigma.reg2, -2) ; sigma.reg2 ~ dunif(0, 5)

# Covariate

cov1 <- c(1,2,3,4,5,6)

cov2 <- c(0,0,0,1,2,3)

cov3 <- c(0,0,0,0,0,1)

# Regression model

for (i in age.unknown){

Trait[i] ~ dnorm(alpha0 + beta4[year[i]] + eps[ID[i]] + gamma

* pop.ID[i], tau.reg1)

}

for (i in age.known){

Trait[i] ~ dnorm(beta0 + beta1 * cov1[AGE[i]]

+ beta2 * cov2[AGE[i]] + beta3 * cov3[AGE[i]] + beta4[year[i]] +

eps[ID[i]] + beta5 * AgeLO[i] + beta6 * AgeFO[i] + gamma *

pop.ID[i],

tau.reg2)

}

# Derived quantities

slope1 <- beta1

slope2 <- beta1 + beta2

slope3 <- beta1 + beta2 + beta3

}

Table S1: Model selection for within individual age-trajectories of five reproductive traits in female pied flycatchers including slope estimates. ΔWAIC is the difference in WAIC (widely applicable information criterion) between a given model and the model with the lowest WAIC. w_i_ is the weight of evidence in favor of model *i* given the set of candidate models. Model notation: Cst = no age effect, Age = categorical age effect, Lin = linear effect of age, Qua = quadratic effect of age, ThrX = single threshold model with a breaking point at age X, ThrX-X’ = double threshold model with breaking points at ages X and X’. Slopes 1 and 2 are the linear and quadratic effects of age for the quadratic model and the linear effect of age before and after the break point for single threshold models. Similarly slope 3 is the linear effect of age after the second threshold for double threshold models. f is the proportion of the posterior with the same sign as the mean. Laying date, Clutch size and Brood size have been standardized so that the slopes can be compared among traits. Slope estimates of nest and egg success are on the logit scale.

| **Laying date** | | | | | | | | | |
| --- | --- | --- | --- | --- | --- | --- | --- | --- | --- |
| **Age (α)** | **WAIC** | **ΔWAIC** | **w_i_** | **Slope 1 [95% CRI]** | **f** | **Slope 2 [95% CRI]** | **f** | **Slope 3 [95% CRI]** | **f** |
| Cst | 3503.0 | 62.8 | 0.00 |  |  |  |  |  |  |
| Age | 3448.4 | 8.2 | 0.01 |  |  |  |  |  |  |
| Lin | 3474.4 | 34.1 | 0.00 | -0.15 [-0.21,-0.08] | 1.00 |  |  |  |  |
| Qua | 3441.5 | 1.2 | 0.20 | -0.60 [-0.80,-0.41] | 1.00 | 0.07 [0.04,0.10] | 1 |  |  |
| Thr2 | 3445.9 | 5.7 | 0.02 | -0.49 [-0.64,-0.34] | 1.00 | -0.04 [-0.12,0.03] | 0.87 |  |  |
| **Thr3** | **3440.2** | **0.0** | **0.37** | **-0.38 [-0.45,-0.25]** | **1.00** | **0.07 [-0.04,0.17]** | **0.90** |  |  |
| Thr4 | 3456.3 | 16.0 | 0.00 | -0.24 [-0.32,-0.16] | 1.00 | 0.14 [-0.02,0.30] | 0.95 |  |  |
| Thr5 | 3464.2 | 23.9 | 0.00 | -0.19 [-0.27,-0.12] | 1.00 | 0.33 [-0.03,0.67] | 0.97 |  |  |
| Thr2-3 | 3441.3 | 1.1 | 0.22 | -0.44 [-0.60,-0.29] | 1.00 | -0.22 [-0.41,-0.03] | 0.99 | 0.04 [-0.07,0.15] | 0.76 |
| Thr2-4 | 3445.6 | 5.4 | 0.03 | -0.47 [-0.62,-0.32] | 1.00 | -0.10 [-0.21,0.02] | 0.95 | 0.05 [-0.11,0.22] | 0.74 |
| Thr2-5 | 3444.7 | 4.4 | 0.04 | -0.49 [-0.64,-0.33] | 1.00 | -0.07 [-0.16,0.03] | 0.92 | 0.11 [-0.24,0.46] | 0.74 |
| Thr3-4 | 3444.2 | 4.0 | 0.05 | -0.36 [-0.47,-0.26] | 1.00 | 0.14 [-0.09,0.37] | 0.89 | 0.01 [-0.16,0.19] | 0.56 |
| Thr3-5 | 3443.8 | 3.6 | 0.06 | -0.36 [-0.46,-0.25] | 1.00 | 0.09 [-0.06,0.24] | 0.88 | -0.02 [-0.40,0.36] | 0.54 |
| Thr4-5 | 3459.0 | 18.8 | 0.00 | -0.24 [-0.32,-0.16] | 1.00 | 0.22 [-0.11,0.53] | 0.91 | 0.03 [-0.38,0.44] | 0.56 |
| **Clutch size** | | | | | | | | | |
| **Age (α)** | **WAIC** | **ΔWAIC** | **w_i_** | **Slope 1 [95% CRI]** | **f** | **Slope 2 [95% CRI]** | **f** | **Slope 3 [95% CRI]** | **f** |
| Cst | 3803.2 | 23.6 | 0.00 |  |  |  |  |  |  |
| Age | 3786.6 | 7.0 | 0.01 |  |  |  |  |  |  |
| Lin | 3802.0 | 22.4 | 0.00 | 0.08 [0.01,0.16] | 0.98 |  |  |  |  |
| **Qua** | **3779.6** | **0.0** | **0.29** | **0.55 [0.33,0.76]** | **1.00** | **-0,07 [-0.11,-0.04]** | **1.00** |  |  |
| Thr2 | 3780.7 | 1.1 | 0.17 | 0.47 [0.29,0.65] | 1.00 | -0,03 [-0.12,0.06] | 0.76 |  |  |
| Thr3 | 3781.3 | 1.7 | 0.13 | 0.28 [0.16,0.40] | 1.00 | -0,12 [-0.24,0.00] | 0.97 |  |  |
| Thr4 | 3787.9 | 8.2 | 0.00 | 0.18 [0.08,0.27] | 1.00 | -0,20 [-0.39,-0.02] | 0.99 |  |  |
| Thr5 | 3796.9 | 17.2 | 0.00 | 0.13 [0.04,0.21] | 1.00 | -0,34 [-0.74,0.05] | 0.96 |  |  |
| Thr2-3 | 3780.8 | 1.2 | 0.16 | 0.45 [0.26,0.63] | 1.00 | 0,07 [-0.17,0.29] | 0.71 | -0.08 [-0.20,0.05] | 0.89 |
| Thr2-4 | 3781.1 | 1.5 | 0.14 | 0.45 [0.26,0.63] | 1.00 | 0,01 [-0.13,0.15] | 0.55 | -0.11 [-0.30,0.09] | 0.86 |
| Thr2-5 | 3783.6 | 4.0 | 0.04 | 0.47 [0.29,0.65] | 1.00 | -0,03 [-0.14,0.08] | 0.70 | -0.06 [-0.46,0.34] | 0.61 |
| Thr3-4 | 3783.9 | 4.2 | 0.04 | 0.29 [0.17,0.41] | 1.00 | -0,17 [-0.45,0.10] | 0.89 | -0.09 [-0.29,0.12] | 0.80 |
| Thr3-5 | 3785.1 | 5.5 | 0.02 | 0.29 [0.17,0.41] | 1.00 | -0,18 [-0.36,0.00] | 0.97 | 0.06 [-0.38,0.50] | 0.61 |
| Thr4-5 | 3792.1 | 12.5 | 0.00 | 0.19 [0.09,0.28] | 1.00 | -0,42 [-0.81,-0.03] | 0.98 | 0.07 [-0.42,0.55] | 0.62 |
| **Brood size** | | | | | | | | | |
| **Age (α)** | **WAIC** | **ΔWAIC** | **w_i_** | **Slope 1 [95% CRI]** | **f** | **Slope 2 [95% CRI]** | **f** | **Slope 3 [95% CRI]** | **f** |
| Cst | 3866.5 | 6.9 | 0.02 |  |  |  |  |  |  |
| Age | 3868.9 | 9.3 | 0.01 |  |  |  |  |  |  |
| Lin | 3869.9 | 10.3 | 0.00 | 0.03 [-0.06,0.11] | 0.74 |  |  |  |  |
| Qua | 3863.4 | 3.8 | 0.09 | 0.33 [0.08,0.58] | 1.00 | -0,05 [-0.09,-0.01] | 0.99 |  |  |
| **Thr2** | **3859.6** | **0.0** | **0.57** | **0.32 [0.12,0.51]** | **1.00** | **-0,06 [-0.16,0.04]** | **0.89** |  |  |
| Thr3 | 3865.7 | 6.1 | 0.03 | 0.15 [0.01,0.28] | 0.98 | -0,10 [-0.23,0.04] | 0.92 |  |  |
| Thr4 | 3871.0 | 11.4 | 0.00 | 0.08 [-0.02,0.18] | 0.93 | -0,13 [-0.34,0.08] | 0.88 |  |  |
| Thr5 | 3868.6 | 9.1 | 0.01 | 0.06 [-0.04,0.15] | 0.88 | -0,28 [-0.72,0.16] | 0.89 |  |  |
| Thr2-3 | 3864.3 | 4.7 | 0.05 | 0.33 [0.13,0.55] | 1.00 | -0,10 [-0.36,0.15] | 0.78 | -0.05 [-0.19,0.10] | 0.74 |
| Thr2-4 | 3862.8 | 3.2 | 0.11 | 0.33 [0.12,0.54] | 1.00 | -0,08 [-0.23,0.08] | 0.84 | -0.04 [-0.26,0.18] | 0.64 |
| Thr2-5 | 3863.2 | 3.7 | 0.09 | 0.32 [0.12,0.52] | 1.00 | -0,06 [-0.18,0.06] | 0.83 | -0.08 [-0.54,0.38] | 0.64 |
| Thr3-4 | 3868.1 | 8.5 | 0.01 | 0.16 [0.02,0.30] | 0.98 | -0,17 [-0.48,0.13] | 0.86 | -0.05 [-0.28,0.18] | 0.65 |
| Thr3-5 | 3868.3 | 8.7 | 0.01 | 0.15 [0.01,0.28] | 0.98 | -0,10 [-0.30,0.10] | 0.84 | -0.08 [-0.57,0.42] | 0.62 |
| Thr4-5 | 3873.4 | 13.8 | 0.00 | 0.08 [-0.03,0.18] | 0.92 | -0,10 [-0.52,0.33] | 0.69 | -0.16 [-0.70,0.39] | 0.71 |
| **Nest success** | | | | | | | | | |
| **Age (α)** | **WAIC** | **ΔWAIC** | **w_i_** | **Slope 1 [95% CRI]** | **f** | **Slope 2 [95% CRI]** | **f** | **Slope 3 [95% CRI]** | **f** |
| Cst | 1395.9 | 1.1 | 0.14 |  |  |  |  |  |  |
| Age | 1403.7 | 8.9 | 0.00 |  |  |  |  |  |  |
| Lin | 1395.5 | 0.7 | 0.17 | -0.30 [-0.61,0.00] | 0.97 |  |  |  |  |
| Qua | 1397.2 | 2.4 | 0.07 | -0.01 [-0.71,0.72] | 0.72 | -0.05 [-0.15,0.06] | 0.81 |  |  |
| Thr2 | 1397.7 | 2.9 | 0.06 | -0.14 [-0.73,0.43] | 0.68 | -0.36 [-0.70,-0.01] | 0.98 |  |  |
| Thr3 | 1398.7 | 3.9 | 0.04 | -0.27 [-0.68,0.15] | 0.90 | -0.35 [-0.78,0.10] | 0.94 |  |  |
| Thr4 | 1397.4 | 2.6 | 0.06 | -0.20 [-0.58,0.16] | 0.86 | -0.58 [-1.19,0.07] | 0.96 |  |  |
| **Thr5** | **1394.8** | **0.0** | **0.24** | **-0.18 [-0.52,0.15]** | **0.85** | **-1.26 [-2.48,0.01]** | **0.97** |  |  |
| Thr2-3 | 1401.2 | 6.4 | 0.01 | -0.09 [-0.70,0.52] | 0.62 | -0.48 [1.24,0.27] | 0.90 | -0.30 [-0.75,0.16] | 0.90 |
| Thr2-4 | 1399.9 | 5.1 | 0.02 | -0.22 [-0.81,0.36] | 0.76 | -0.20 [-0.71,0.30] | 0.78 | -0.60 [-1.24,0.04] | 0.96 |
| Thr2-5 | 1397.9 | 3.1 | 0.05 | -0.23 [-0.81,0.33] | 0.78 | -0.15 [-0.59,0.30] | 0.75 | -1.33 [-2.68,0.02] | 0.97 |
| Thr3-4 | 1399.3 | 4.5 | 0.03 | -0.34 [-0.77,0.09] | 0.94 | 0.44 [-0.61,1.61] | 0.78 | -0.84 [-1.60,-0.11] | 0.98 |
| Thr3-5 | 1397.2 | 2.4 | 0.07 | -0.34 [-0.75,0.08] | 0.95 | 0.32 [-0.46,1.18] | 0.77 | -2.04 [-3.85,-0.39] | 0.99 |
| Thr4-5 | 1398.7 | 3.9 | 0.03 | -0.24 [-0.60,0.12] | 0.90 | 1.25 [-0.87,4.33] | 0.84 | -2.56 [-5.73,-0.30] | 0.99 |
| **Egg success** | | | | | | | | | |
| **Age (α)** | **WAIC** | **ΔWAIC** | **w_i_** | **Slope 1 [95% CRI]** | **f** | **Slope 2 [95% CRI]** | **f** | **Slope 3 [95% CRI]** | **f** |
| **Cst** | **3312.5** | **0.0** | **0.53** |  |  |  |  |  |  |
| Age | 3325.8 | 13.3 | 0.00 |  |  |  |  |  |  |
| Lin | 3314.8 | 2.4 | 0.16 | -0.08 [-0.18,0.02] | 0.93 |  |  |  |  |
| Qua | 3317.0 | 4.5 | 0.06 | -0.03 [-0.37,0.30] | 0.58 | -0.01 [-0.06,0.04] | 0.61 |  |  |
| Thr2 | 3316.6 | 4.1 | 0.07 | 0.00 [-0.27,0.27] | 0.51 | -0.10 [-0.22,0.03] | 0.94 |  |  |
| Thr3 | 3317.8 | 5.3 | 0.04 | -0.07 [-0.25,0.11] | 0.79 | -0.08 [-0.25,0.09] | 0.83 |  |  |
| Thr4 | 3318.0 | 5.6 | 0.03 | -0.08 [-0.21,0.06] | 0.87 | -0.09 [-0.34,0.17] | 0.74 |  |  |
| Thr5 | 3316.9 | 4.5 | 0.06 | -0.06 [-0.18,0.06] | 0.84 | -0.21 [-0.76,0.34] | 0.77 |  |  |
| Thr2-3 | 3320.7 | 8.3 | 0.01 | 0.02 [-0.27,0.30] | 0.56 | -0.19 [-0.53,0.13] | 0.87 | -0.06 [-0.24,0.12] | 0.75 |
| Thr2-4 | 3320.9 | 8.5 | 0.01 | 0.01 [-0.27,0.29] | 0.54 | -0.13 [-0.33,0.07] | 0.90 | -0.06 [-0.33,0.22] | 0.66 |
| Thr2-5 | 3321.3 | 8.9 | 0.01 | 0.00 [-0.26,0.27] | 0.50 | -0.09 [-0.25,0.07] | 0.86 | -0.17 [-0.74,0.40] | 0.72 |
| Thr3-4 | 3321.1 | 8.6 | 0.01 | -0.07 [-0.26,0.11] | 0.78 | -0.08 [-0.46,0.30] | 0.67 | -0.08 [-0.37,0.20] | 0.72 |
| Thr3-5 | 3318.9 | 6.4 | 0.02 | -0.08 [-0.25,0.10] | 0.82 | -0.03 [-0.27,0.22] | 0.59 | -0.25 [-0.86,0.35] | 0.79 |
| Thr4-5 | 3321.6 | 9.1 | 0.01 | -0.08 [-0.22,0.06] | 0.88 | 0.12 [-0.42,0.67] | 0.67 | -0.35 [-1.01,0.33] | 0.85 |

Table S2: Model selection for population level age-trajectories of five reproductive traits in female pied flycatchers including slope estimates. ΔWAIC is the difference in WAIC (widely applicable information criterion) between a given model and the model with the lowest WAIC. w_i_ is the weight of evidence in favor of model i given the set of candidate models. Model notation: Cst = no age effect, Age = fixed age effect, Lin = linear effect of age, Qua = quadratic effect of age, ThrX = single threshold model with a breaking point at age X, ThrX-X’ = double threshold model with breaking points at ages X and X’. Slopes 1 and 2 are the linear and quadratic effects of age for the quadratic model and the linear effect of age before and after the breaking point for single threshold models. Similarly slope 3 is the linear effect of age after the second threshold for double threshold models. f is the proportion of the posterior with the same sign as the mean. Laying date, Clutch size and Brood size have been standardized so that the slopes can be compared among traits. Slope estimates of nest and egg success are on the logit scale.

| **Laying date** | | | | | | | | | |
| --- | --- | --- | --- | --- | --- | --- | --- | --- | --- |
| **Age (α)** | **WAIC** | **ΔWAIC** | **w_i_** | **Slope 1 [95% CRI]** | **f** | **Slope 2 [95% CRI]** | **f** | **Slope 3 [95% CRI]** | **f** |
| Cst | 3646.2 | 69.2 | 0.00 |  |  |  |  |  |  |
| Age | 3584.5 | 7.4 | 0.01 |  |  |  |  |  |  |
| Lin | 3597.0 | 19.9 | 0.00 | -0.18 [-0.23,-0.13] | 1.00 |  |  |  |  |
| Qua | 3578.6 | 1.6 | 0.15 | -0.61 [-0.81,-0.41] | 1.00 | 0.07 [0.04,0.10] | 1.00 |  |  |
| Thr2 | 3579.7 | 2.6 | 0.09 | -0.52 [-0.68,0.36] | 1.00 | -0.07 [-0.14,0.00] | 0.97 |  |  |
| **Thr3** | **3577.0** | **0.0** | **0.34** | **-0.37 [-0.47,-0.27]** | **1.00** | **0.04 [-0.07,0.14]** | **0.76** |  |  |
| Thr4 | 3588.0 | 11.0 | 0.00 | -0.26 [-0.34,-0.19] | 1.00 | 0.10 [-0.07,0.27] | 0.86 |  |  |
| Thr5 | 3593.2 | 16.2 | 0.00 | -0.22 [-0.29,-0.16] | 1.00 | 0.28 [-0.09,0.64] | 0.93 |  |  |
| Thr2-3 | 3578.5 | 1.4 | 0.17 | -0.46 [-0.63,-0.29] | 1.00 | -0.25 [-0.46,-0.04] | 0.99 | 0.01 [-0.10,0.12] | 0.58 |
| Thr2-4 | 3581.1 | 4.1 | 0.05 | -0.50 [-0.66,0.34] | 1.00 | -0.12 [-0.23,0.00] | 0.97 | 0.02[-0.16,0.19] | 0.57 |
| Thr2-5 | 3581.8 | 4.8 | 0.03 | -0.51 [-0.67,-0.34] | 1.00 | -0.09 [-0.18,0.00] | 0.97 | 0.06 [-0.32,0.45] | 0.62 |
| Thr3-4 | 3579.8 | 2.8 | 0.08 | -0.39 [-0.49,-0.29] | 1.00 | 0.15[-0.11,0.40] | 0.87 | -0.03 [-0.22,0.15] | 0.63 |
| Thr3-5 | 3580.1 | 3.1 | 0.07 | -0.38 [-0.48,-0.28] | 1.00 | 0.08 [-0.09,0.24] | 0.83 | -0.09 [-0.49,0.32] | 0.66 |
| Thr4-5 | 3592.1 | 15.1 | 0.00 | -0.27 [-0.34,-0.20] | 1.00 | 0.20 [-0.17,0.57] | 0.86 | -0.03 [-0.48,0.43] | 0.56 |
| **Clutch size** | | | | | | | | | |
| **Age (α)** | **WAIC** | **ΔWAIC** | **w_i_** | **Slope 1 [95% CRI]** | **f** | **Slope 2 [95% CRI]** | **f** | **Slope 3 [95% CRI]** | **f** |
| Cst | 3877.3 | 40.9 | 0.00 |  |  |  |  |  |  |
| Age | 3845.9 | 9.5 | 0.00 |  |  |  |  |  |  |
| Lin | 3855.3 | 18.9 | 0.00 | 0.14 [0.08,0.19] | 1.00 |  |  |  |  |
| Qua | 3841.6 | 5.2 | 0.04 | 0.56 [0.33,0.77] | **1**.00 | -0.07 [-0.10,-0.03] | 1.00 |  |  |
| **Thr2** | **3836.4** | **0.0** | **0.54** | **0.52 [0.34,0.69]** | **1.00** | **0.02 [-0.06,0.09]** | **0.66** |  |  |
| Thr3 | 3841.8 | 5.5 | 0.04 | 0.32 [0.21,0.42] | 1.00 | -0.06 [-0.17,0.05] | 0.85 |  |  |
| Thr4 | 3849.7 | 13.3 | 0.00 | 0.22 [0.14,0.30] | 1.00 | -0.12 [-0.30,0.07] | 0.89 |  |  |
| Thr5 | 3856.0 | 19.6 | 0.00 | 0.17 [0.11,0.24] | 1.00 | -0.20 [-0.59,0.19] | 0.84 |  |  |
| Thr2-3 | 3839.0 | 2.6 | 0.15 | 0.50 [0.32,0.69] | 1.00 | 0.08 [-0.16,0.31] | 0.75 | -0.01 [-0.13,0.11] | 0.57 |
| Thr2-4 | 3839.3 | 2.9 | 0.12 | 0.52 [0.34,0.70] | 1.00 | 0.03 [-0.10,0.16] | 0.67 | -0.01 [-0.20,0.18] | 0.35 |
| Thr2-5 | 3840.3 | 4.0 | 0.08 | 0.53 [0.35,0.71] | 1.00 | 0.00 [-0.11,0.11] | 0.50 | 0.10 [-0.31,0.51] | 0.68 |
| Thr3-4 | 3843.3 | 6.9 | 0.02 | 0.33 [0.22,0.44] | 1.00 | -0.17 [-0.46,0.12] | 0.87 | 0.01 [-0.19,0.22] | 0.54 |
| Thr3-5 | 3844.8 | 8.4 | 0.01 | 0.34 [0.23,0.44] | 1.00 | -0.16 [-0.34,0.03] | 0.95 | 0.22 [-0.22,0.67] | 0.83 |
| Thr4-5 | 3851.4 | 15.0 | 0.00 | 0.23 [0.15,0.31] | 1.00 | -0.40 [-0.82,0.02] | 0.97 | 0.24 [-0.27,0.75] | 0.82 |
| **Brood size** | | | | | | | | | |
| **Age (α)** | **WAIC** | **ΔWAIC** | **w_i_** | **Slope 1 [95% CRI]** | **f** | **Slope 2 [95% CRI]** | **f** | **Slope 3 [95% CRI]** | **f** |
| Cst | 3913.8 | 9.3 | 0.00 |  |  |  |  |  |  |
| Age | 3913.9 | 9.4 | 0.00 |  |  |  |  |  |  |
| Lin | 3911.6 | 7.0 | 0.01 | 0.06 [-0.00,0.11] | 0.97 |  |  |  |  |
| Qua | 3908.5 | 4.0 | 0.07 | 0.35 [0.11,0.60] | 1.00 | -0.05 [-0.09,-0.01] | 0.99 |  |  |
| **Thr2** | **3904.5** | **0.0** | **0.50** | **0.34 [0.14,0.54]** | **1.00** | **-0.04 [-0.12,0.05]** | **0.80** |  |  |
| Thr3 | 3908.5 | 4.0 | 0.07 | 0.17 [0.05,0.29] | 1.00 | -0.07 [-0.20,0.06] | 0.86 |  |  |
| Thr4 | 3911.1 | 6.6 | 0.02 | 0.10 [0.02,0.19] | 0.99 | -0.09 [-0.30,0.11] | 0.82 |  |  |
| Thr5 | 3912.1 | 7.6 | 0.01 | 0.09 [0.01,0.16] | 0.99 | -0.25 [-0.70,0.19] | 0.87 |  |  |
| Thr2-3 | 3907.9 | 3.4 | 0.09 | 0.34 [0.11,0.54] | 1.00 | -0.05 [-0.30,0.21] | 0.64 | -0.03 [-0.16,0.11] | 0.65 |
| Thr2-4 | 3907.8 | 3.3 | 0.10 | 0.34 [0.14,0.55] | 1.00 | -0.05 [-0.19,0.10] | 0.74 | -0.01 [-0.22,0.20] | 0.54 |
| Thr2-5 | 3907.9 | 3.4 | 0.09 | 0.33 [0.13,0.54] | 1.00 | -0.03 [-0.14,0.09] | 0.66 | -0.08 [-0.53,0.39] | 0.63 |
| Thr3-4 | 3911.5 | 7.0 | 0.02 | 0.18 [0.06,0.31] | 1.00 | -0.16 [-0.48,0.15] | 0.84 | -0.01 [-0.24,0.21] | 0.54 |
| Thr3-5 | 3913.0 | 8.5 | 0.01 | 0.17 [0.05,0.29] | 1.00 | -0.07 [-0.27,0.13] | 0.76 | -0.07 [-0.57,0.42] | 0.60 |
| Thr4-5 | 3915.5 | 11.0 | 0.00 | 0.10 [0.01,0.19] | 0.99 | -0.05 [-0.48,0.38] | 0.59 | -0.15 [-0.69,0.39] | 0.71 |
| **Nest success** | | | | | | | | | |
| **Age (α)** | **WAIC** | **ΔWAIC** | **w_i_** | **Slope 1 [95% CRI]** | **f** | **Slope 2 [95% CRI]** | **f** | **Slope 3 [95% CRI]** | **f** |
| Cst | 1406.9 | 1.2 | 0.15 |  |  |  |  |  |  |
| Age | 1414.3 | 8.5 | 0.00 |  |  |  |  |  |  |
| Lin | 1407.5 | 1.8 | 0.11 | 0.14 [-0.03,0.31] | 0.95 |  |  |  |  |
| Qua | 1408.9 | 3.2 | 0.06 | 0.00 [-0.16,1.17] | 0.93 | -0.06 [-0.17,0.05] | 0.87 |  |  |
| Thr2 | 1409.7 | 4.0 | 0.04 | 0.33 [-0.16,0.84] | 0.90 | 0.07 [-0.17,0.32] | 0.70 |  |  |
| Thr3 | 1410.3 | 4.6 | 0.03 | 0.22 [-0.08,0.54] | 0.92 | 0.04 [-0.32,0.42] | 0.57 |  |  |
| Thr4 | 1408.0 | 2.3 | 0.09 | 0.26 [0.03,0.51] | 0.99 | -0.25 [-0.81,0.35] | 0.81 |  |  |
| **Thr5** | **1405.7** | **0.0** | **0.28** | **0.28 [0.07,0.50]** | **1.00** | **-1.06 [-2.20,0.15]** | **0.96** |  |  |
| Thr2-3 | 1413.1 | 7.4 | 0.01 | 0.33 [-0.21,0.85] | 0.88 | 0.07 [-0.59,0.80] | 0.57 | 0.07 [-0.31,0.47] | 0.63 |
| Thr2-4 | 1411.2 | 5.5 | 0.02 | 0.25 [-0.27,0.76] | 0.83 | 0.28 [-0.15,0.73] | 0.90 | -0.26 [-0.88,0.37] | 0.80 |
| Thr2-5 | 1408.6 | 2.9 | 0.06 | 0.22 [-0.29,0.74] | 0.80 | 0.31 [-0.03,0.71] | 0.96 | -1.13 [-2.47,0.20] | 0.95 |
| Thr3-4 | 1410.4 | 4.6 | 0.03 | 0.14 [-0.17,0.47] | 0.80 | 0.90 [-0.13,2.08] | 0.95 | -0.50[-1.24,0.19] | 0.92 |
| Thr3-5 | 1408.0 | 2.3 | 0.09 | 0.14 [-0.18,0.46] | 0.80 | 0.75 [0.01,0.63] | 0.98 | -1.78[-3.60,-0.13] | 0.98 |
| Thr4-5 | 1409.7 | 3.9 | 0.04 | 0.23 [-0.01,0.48] | 0.97 | 1.60 [-0.44,4.88] | 0.92 | -2.26 [-5.63,-0.04] | 0.98 |
| **Egg success** | | | | | | | | | |
| **Age (α)** | **WAIC** | **ΔWAIC** | **w_i_** | **Slope 1 [95% CRI]** | **f** | **Slope 2 [95% CRI]** | **f** | **Slope 3 [95% CRI]** | **f** |
| **Cst** | **3419.1** | **0.0** | **0.63** |  |  |  |  |  |  |
| Age | 3435.3 | 16.3 | 0.00 |  |  |  |  |  |  |
| Lin | 3421.9 | 2.8 | 0.15 | -0.03 [-0.09,0.04] | 0.80 |  |  |  |  |
| Qua | 3424.4 | 5.3 | 0.04 | 0.10 [-0.16,0.38] | 0.62 | -0.02 [-0.06,0.02] | 0.84 |  |  |
| Thr2 | 3424.1 | 5.0 | 0.05 | 0.10 [-0.12,0.32] | 0.81 | -0.07 [-0.15,0.03] | 0.92 |  |  |
| Thr3 | 3425.8 | 6.7 | 0.02 | 0.02 [-0.12,0.15] | 0.60 | -0.07 [-0.21,0.07] | 0.85 |  |  |
| Thr4 | 3425.2 | 6.1 | 0.03 | -0.01 [-0.10,0.09] | 0.56 | -0.09 [-0.30,0.14] | 0.78 |  |  |
| Thr5 | 3424.6 | 5.5 | 0.04 | 0.00 [-0.08,0.09] | 0.52 | -0.29 [-0.74,0.18] | 0.89 |  |  |
| Thr2-3 | 3428.3 | 9.2 | 0.01 | 0.12 [-0.12,0.36] | 0.84 | -0.11[-0.39,0.17] | 0.78 | -0.05 [-0.19,0.10] | 0.74 |
| Thr2-4 | 3428.5 | 9.4 | 0.01 | 0.10 [-0.14,0.33] | 0.80 | -0.07 [-0.22,0.09] | 0.81 | -0.05 [-0.28,0.18] | 0.68 |
| Thr2-5 | 3428.1 | 9.0 | 0.01 | 0.08 [-0.14,0.29] | 0.75 | -0.03[-0.16,0.10] | 0.68 | -0.24 [-0.72,0.24] | 0.83 |
| Thr3-4 | 3429.9 | 10.8 | 0.00 | 0.02 [-0.13,0.16] | 0.59 | -0.07 [-0.40,0.27] | 0.65 | -0.07 [-0.30,0.17] | 0.72 |
| Thr3-5 | 3429.0 | 9.9 | 0.00 | 0.00 [-0.14,0.14] | 0.51 | 0.01 [-0.21,0.23] | 0.53 | -0.30 [-0.82,0.23] | 0.87 |
| Thr4-5 | 3428.7 | 9.6 | 0.01 | -0.02 [-0.11,0.08] | 0.63 | 0.18 [-0.30,0.69] | 0.76 | -0.42 [-1.02,0.17] | 0.91 |

Figure S1 : Estimated average within-individual age trajectories in clutch size in females pied flycatchers after accounting for within-year variation in laying date. Left panel: points and vertical bars show estimates from a model with age fitted as a categorical variable, ± standard error. The solid black line shows the mean aging pattern predicted by the model with the grey-shaded area showing standard errors around this prediction. Numbers at the top refer to sample sizes (number of individuals of a given age). Right panel: posterior distribution of the linear (slope 1) and quadratic (slope 2) estimates of the quadratic effect of age.


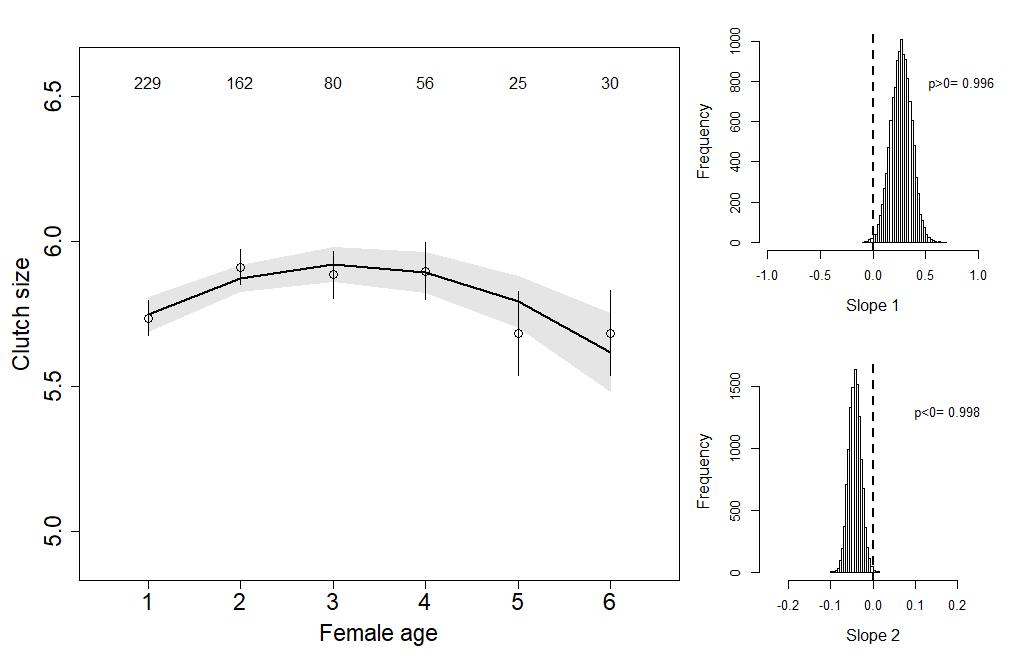


Figure S2 : Estimated average within-individual age trajectories in brood size in females pied flycatcher after accounting for within-year variation in laying date. Left panel: points and vertical bars show estimates from a model with age fitted as a categorical variable, ± standard error. The solid black line shows the mean aging pattern predicted by the model with the grey-shaded area showing standard errors around this prediction. Numbers at the top refer to sample sizes (number of individuals of a given age). Right panel: posterior distribution of the two linear effect of age before (slope 1) and after (slope 2) the breaking point


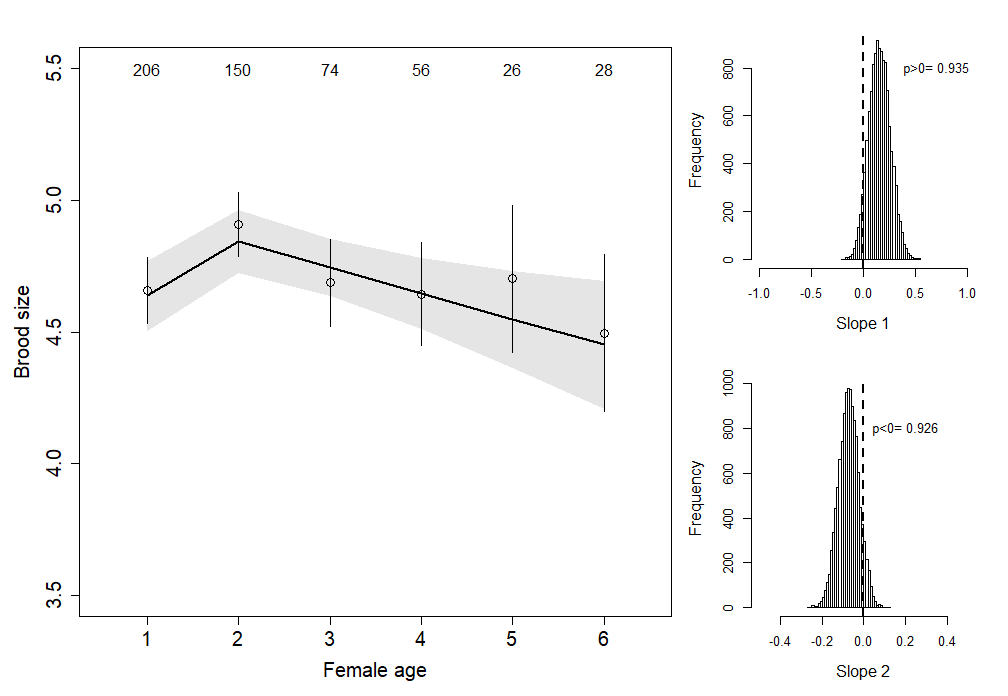

Supplement: Supplementary file 1 — Supplementary file1 (DOCX 142 kb) [file 442_2021_4963_MOESM1_ESM.docx]
